# Double Digest: A Rare Case Report of Amphicrine Gastric Carcinoma Co-occurring With Papillary Thyroid Carcinoma

**DOI:** 10.7759/cureus.59205

**Published:** 2024-04-28

**Authors:** Dominique M Ebedes, Samer Ganam, Joseph A Sujka, Christopher G DuCoin

**Affiliations:** 1 Surgery, University of South Florida Morsani College of Medicine, Tampa, USA; 2 Surgery, Tampa General Hospital, Tampa, USA; 3 General, Bariatric, Foregut, and Hernia Surgery, Tampa General Hospital, Tampa, USA

**Keywords:** gastrectomy, : bariatric surgery, : papillary thyroid carcinoma, amphicrine tumor, gastric cancer (gc)

## Abstract

With improved cancer treatments and patient lifespans, the incidence of a second cancer diagnosis in a person’s lifetime is increasing. While dual cancer diagnoses during one’s lifetime are becoming more common, diagnosis with two separate cancers simultaneously is less so. In this report we present a 55-year-old obese woman with a history of chronic lymphocytic thyroiditis and a non-specific family history of thyroid cancer who received synchronous diagnoses of amphicrine carcinoma (AC) and papillary thyroid carcinoma (PTC) during work-up for bariatric surgery. AC is a very rare form of gastric cancer characterized by the presence of both endocrine and epithelial cell components within the same cell with only a few case reports in the literature. This is the first case report to present the co-occurrence of AC with PTC.

## Introduction

Gastric cancer

Gastric cancer is one of the most prevalent malignancies worldwide, ranking as the fourth leading cause of cancer death globally [1]. It has a poor prognosis with a five-year survival rate of 20% [1]. This high level of aggressiveness combined with the heterogeneity of gastric cancer make it a major threat to global health. Gastric amphicrine carcinoma (AC) is an exceedingly rare form of gastric carcinoma that is a sub-type of mixed neuroendocrine and non-neuroendocrine neoplasms (MiNENs). The majority of MiNENs are biphasic tumors in which both the endocrine and non-neuroendocrine components are morphologically and immunophenotypically distinct. Amphicrine carcinomas are unique in that there is dual differentiation with neuroendocrine and exocrine or mucin granules within a single cell. These lesions are challenging both from a diagnostic and therapeutic standpoint, as there is little research on AC given its rarity. To date there has not been a reported case of concomitant gastric AC and papillary thyroid carcinoma (PTC). Previously reported gastric AC typically occurs in males in their 60s. Common treatments included total gastrectomy [2] and wedge resection [3] typically resulting in lack of recurrence at six-month follow-up.

Gastric cancer and the thyroid

Reports have shown an association between thyroid diseases and risk factors for gastric carcinoma, such as *H. pylori *[4]. Furthermore, the incidence of goiter and autoimmune thyroid disease has been shown to be higher in patients with gastric carcinoma [4].

## Case presentation

**Figure 1:**
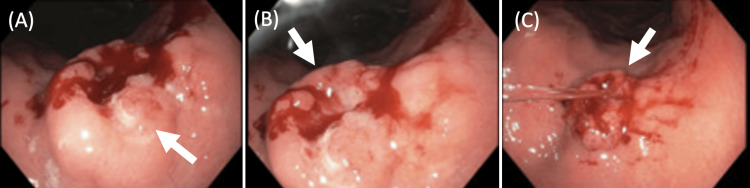
Pre-bariatric surgery endoscopy demonstrating gastric ulcer on the lesser curvature of the stomach. Panels A, B, and C demonstrate the ulcer (indicated by the white arrow) from three different perspectives.

An asymptomatic 55-year-old woman presented for a bariatric surgery consultation. Her medical history included morbid obesity class 4, obstructive sleep apnea (OSA), myocardial infarction (MI), hypertension (HTN), status post *H. pylori *gastritis, and suspected lymphocytic thyroiditis. Her family history was significant for HTN and heart failure, as well as a history of thyroid cancer in the family (relationship and type unknown). Upon routine esophagogastroduodenoscopy (EGD) prior to bariatric surgery, an incidental gastric ulcer was found at the lesser curvature of the upper gastric body (Figure 1). Five non-bleeding cratered ulcers with no stigmata of bleeding were found on the lesser curvature with the largest lesion measuring 5 mm in greatest dimension. No gross lesions were noted in the esophagus. The Z-line, the junction that delineates the stratified squamous epithelium in the esophagus from the intestinal epithelium of the gastric cardia, appeared regular.

**Figure 2:**
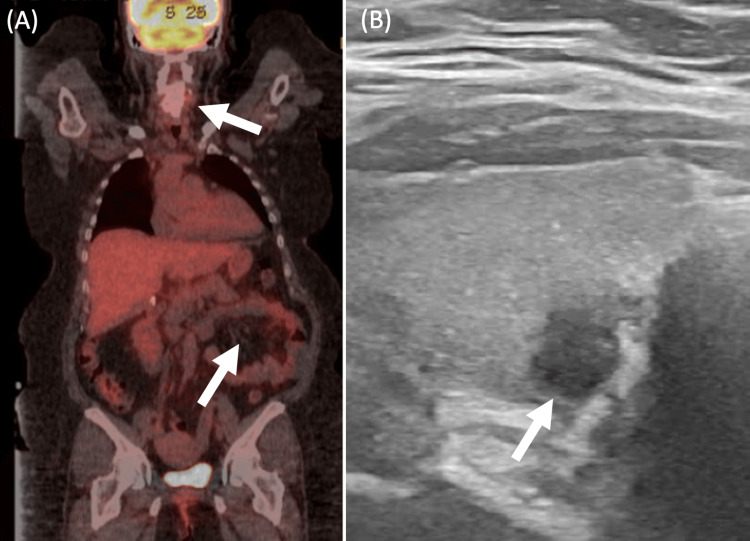
Imaging work-up for suspected gastric cancer demonstrates diffuse gastric uptake on positron emission tomography (PET) CT and a thyroid nodule on PET CT and ultrasound (US). Panel A shows the patient's full body PET CT scan with non-specific diffuse uptake in the stomach and right lobe of the thyroid. Panel B is a follow-up thyroid US revealing a hypoechoic solid nodule with indistinct margins and a widened shape measuring 0.7x0.7x0.6 cm in the posterior interpolar right thyroid.

The gastric ulcer biopsy suggested gastric adenocarcinoma. A positron emission tomography (PET) CT was performed, which demonstrated a mild non-specific diffuse uptake in the stomach without hypermetabolic masses, nodules or lymphadenopathy (Figure 2A). Additionally, an asymmetric increase in contrast uptake was observed in the right lobe of the thyroid, which was confirmed as a thyroid mass on ultrasound (Figure 2B). Overall, the patient's findings were consistent with T1b early-stage gastric cancer with no obvious evidence of metastasis and a thyroid mass. Her case was presented at the tumor board, where it was determined that she would benefit most from immediate surgery to remove her gastric tumor.

Surgical management

The patient consented to undergo intraoperative upper endoscopy and robotic-assisted laparoscopic total gastrectomy with roux en Y reconstruction. The EGD showed gastritis and a 2.6 cm tumor along the lesser curvature of the stomach. Robot-assisted investigation of the abdominal cavity showed no evidence of additional disease. Robotic-assisted laparoscopic total gastrectomy was then performed in the typical fashion with roux en Y reconstruction. Her postoperative course was unremarkable, and she was discharged on postoperative day 3.

Pathology

**Figure 3:**
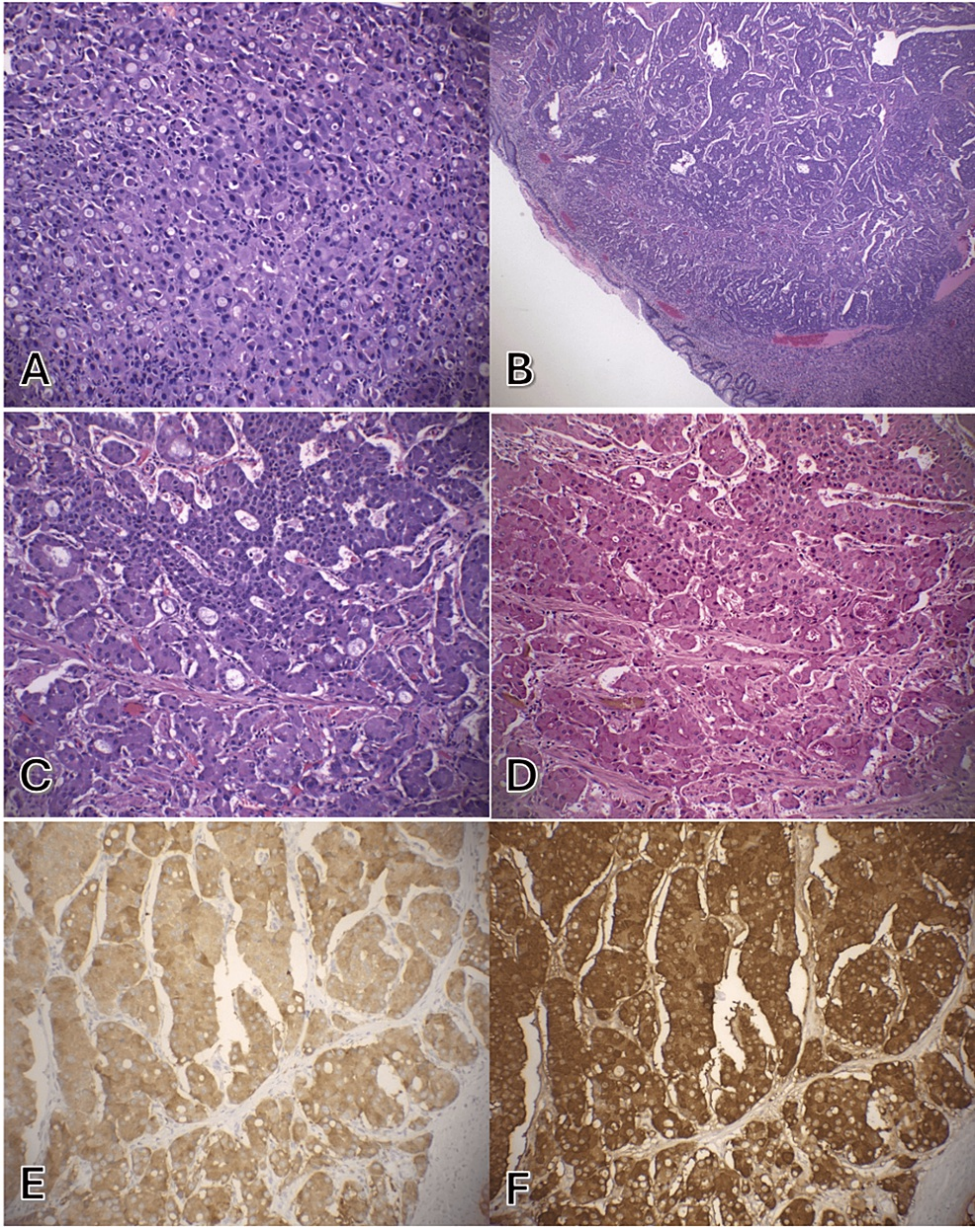
Gastric biopsy specimen consistent with amphicrine carcinoma. Initial biopsy with predominance of glandular morphology without neuroendocrine features (A). Resection with well-differentiated neuroendocrine tumor cells arrayed in nested and glandular patterns (B, C) with intracytoplasmic mucin vacuoles highlighted by mucicarmine (D). The tumor cells are diffusely positive for synaptophysin (D) and chromogranin (E). (hematoxylin-eosin, original magnification x400 [B] and x200 [A, C]; original magnification x200 [D, E, F]).

Grossly, the specimen was a 2.6 cm centrally-ulcerated mass. Histologic examination revealed malignant cells with abundant granular amphophilic cytoplasm, round nuclei, and a finely granular chromatin. Additionally, intracytoplasmic mucin production was observed (Figure 3). The tumor was found to extend from the mucosal surface to the muscularis propria, and there was focal angioinvasion and perineural invasion. The background gastric mucosa was negative for *H. pylori* infection.

Immunohistochemical staining was positive for pankeratin, synaptophysin, chromogranin, and CDX-2 (Figure 3B). A Mucicarmine stain was performed to highlight the presence of mucin. This intracytoplasmic mucin production in cells expressing neuroendocrine markers helped distinguish the mass as an amphicrine carcinoma rather than a neuroendocrine tumor. The tumor was negative for S100, CD117, DOG-1, PAX-8, TTF-1, and gastrin. A Ki67 demonstrated a proliferation index of up to 5% with a mitotic count of two per 2 mm².

Genetic analysis of the gastric tumor specimen revealed mutations in HRAS (variant p.G13R, DNA change c.27G>C, frequency 14.9%, 644 reads, chromosome 11, position 534296) and RAD50 (variant pA149Gfs*5, DNA change c.445dupG, frequency 7.6%, 938 reads, chromosome 5, position 131915088).

Follow-up

**Figure 4:**
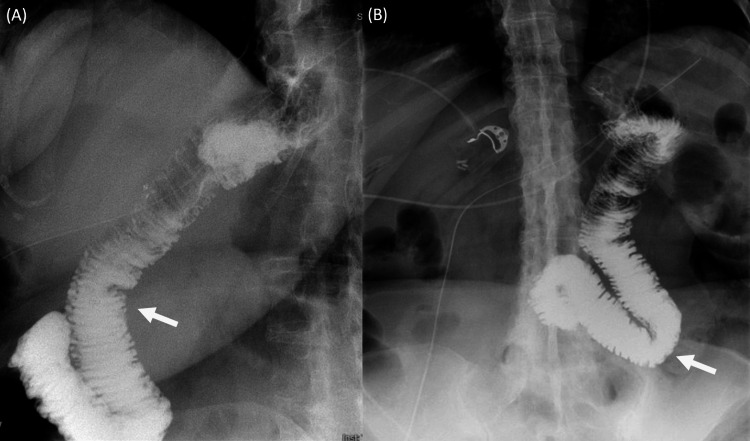
Post-gastrectomy upper GI X-ray series with barium swallow shows anastomosed bowel without evidence of a leak. Panel A corresponds to the lateral X-ray. Panel B corresponds to the anterior-posterior X-ray. The white arrows indicate barium.

There were no postoperative complications, and follow-up fluoroscopic upper gastrointestinal (GI) series demonstrated contrast coursing through the distal esophagus directly into the anastomosed bowel without evidence of a leak (Figure 4). Furthermore, the esophagus was of normal contour, caliber, and motility with no evidence of mucosal abnormality. 

The thyroid nodule

After the incidental discovery of the thyroid mass on PET CT, ultrasound (US) was performed to further characterize the mass. Thyroid ultrasound revealed a markedly hypoechoic solid nodule with indistinct margins and a widened shape measuring 0.7 x 0.7 x 0.6 cm in the posterior interpolar right thyroid, corresponded to a Thyroid Imaging Reporting and Data System (TI-RADS) score of 5. These guidelines suggest a one-year follow-up, however, given the avidity of the nodule on the PET scan, ultrasound-guided biopsy was pursued. 

The patient underwent fine needle aspiration (FNA) of the right thyroid nodule two days prior to her gastric surgery. The results of the FNA were consistent with PTC. The patient underwent a right lobectomy and isthmectomy with central neck dissection approximately a month following her gastric surgery. Histological analysis confirmed PTC, tall cell subtype, measuring 1.1cm, pT1bN0. The background thyroid parenchyma showed lymphocytic thyroiditis, and laboratory studies revealed an increased level of thyroglobulin antibody (104 IU/mL, normal is <1 IU/mL). These findings were consistent with the known association between lymphocytic thyroiditis [5].

## Discussion

On the rarity of gastric amphicrine carcinoma and what we know

Gastric amphicrine carcinoma is an exceedingly rare form of gastric cancer with the first report on PubMed occurring in 1986 and less than 30 papers published since that point. Prior research primarily focuses on case reports and the genetic basis and histology of AC.

The most recently published case report found prior to ours was in a 63-year-old male patient with a gastric nodule that was endoscopically biopsied, revealing histological features of a type 3 well-differentiated gastric neuroendocrine tumor (NET) [3]. The patient then underwent a wedge resection of the gastric wall, which led to a final pathological diagnosis of amphicrine carcinoma with pancreatic acinar cell and neuroendocrine features (pT1b). Hotspot-targeted deep sequencing of 57 genes showed no somatic mutation in agreement with the low mutational burden reported for gastric amphicrine carcinomas. No additional oncological treatment was administered, and the patient remained disease-free after 18 months.

Another recent case report with some parallels to our case described a woman in her 60s with anemia who underwent an upper endoscopy, followed by histopathological examination of biopsy specimens, and was found to have gastric AC with a tumor measuring 5.0 cm in greatest dimension. Similarly, to our patient, this patient was found to have AC incidentally on endoscopy and was successfully treated with a total gastrectomy [2]. Our search yielded no cases that presented a co-diagnosis of thyroid malignancy.

On the co-occurrence of papillary thyroid carcinoma with gastric amphicrine carcinoma

The stomach and thyroid share several morphological and functional similarities. One of these is the ability to concentrate iodides using a membrane-active transport. A 1993 paper theorized a relationship between gastric cancer and excess or decreased iodine levels, which has also been associated with goiter and autoimmune thyroid disease [6]. Furthermore, studies have shown a significant difference in the incidence of autoimmune thyroid disease in patients with gastric carcinoma (27.8%) compared to healthy control subjects (10.9%) [6].

Notably these studies mostly discussed a relationship between gastric carcinoma and autoimmune thyroid diseases. The background lymphocytic thyroiditis on the patient’s thyroid biopsy and the increased level of thyroglobulin antibody (104 IU/mL) suggests that perhaps there was an underlying autoimmune attack of the patient’s thyroid preceding or concomitant with her PTC. Perhaps it is this autoimmune attack against the thyroid that is linked to the patient’s gastric AC diagnosis, rather than her PTC diagnosis. Furthermore, it is notable to mention that these studies examined gastric carcinoma in general (so mostly adenocarcinoma) and not AC. It would be unlikely such a study could be performed with gastric AC given its rarity.

Genetic analysis

Genetic analysis of the patient’s gastric tumor showed two clinically pertinent mutations: (1) an HRAS mutation (variant p.G13R, DNA change c.27G>C, frequency 14.9%, 644 reads, chromosome 11, position 534296) and (2) a RAD50 mutation (variant pA149Gfs*5, DNA change c.445dupG, frequency 7.6%, 938 reads, chromosome 5, position 131915088). The HRAS mutation is particularly pertinent as it has been strongly associated with gastric carcinoma, as well as thyroid carcinoma, including PTC [7,8]. This offers a potential genetic explanation for this patient’s dual cancer diagnoses.

In 2021 a high-resolution copy number (CN) profiling and whole exome sequencing (WES) study was performed on tissue samples from eight gastric amphicrine carcinomas [9]. The study observed a gain of 20q13.12-20q13.2 in five of the eight samples, as well as amplifications of MYT1, NTSR1, and ZBTB46 located in this region as demonstrated by quantitative PCR (qPCR) and immunohistochemistry. These mutations were not noted in our patient’s genetic analysis. More concomitant AC and PTC cases would be needed to establish a potential association with HRAS mutations.

The thyroid tumor as an ‘incidentaloma’

As the quantity and quality of radiographic imaging increases, so does the number of ‘incidentalomas’ (asymptomatic masses discovered upon imaging for another condition) found on imaging [10]. Incidental thyroid masses are found in around 40% of patients who undergo thyroid ultrasounds.

Given the slow-growing nature of PTC and its relatively high prevalence, the association between our patient’s PTC with her AC diagnosis may ultimately be a result of the patient receiving a CT scan that could identify her mass rather than a temporal, genetic, or physiological link between the two diagnoses.

## Conclusions

AC is a rare, emerging form of gastric cancer that poses diagnostic and therapeutic challenges given its rarity. In this report, we presented the first reported case of gastric AC co-occuring with PTC. As of now, there is insufficient evidence to suggest a strong association between PTC and AC, and the detection of gastric AC does not necessarily warrant the search for a thyroid lesion. Further research is needed to better understand gastric AC and determine whether there may be a connection to thyroid pathology, such as PTC. Ultimately this patient's dual cancer diagnoses were likely a result of her significant inflammatory risk factors for thyroid and gastric cancers, including *H. pylori *gastritis and chronic thyroiditis, and the thorough work-up she received. This case highlights the importance of physicians being aware of the possibility of concomitant endocrine tumors and the importance of conducting thorough clinical investigations.

## References

[1] Ilic M, Ilic I (2022). Epidemiology of stomach cancer. World J Gastroenterol.

[2] Hanamatsu Y, Saigo C, Asano N, Kito Y, Nakada K, Takeda Y, Takeuchi T (2019). A case of gastric amphicrine signet-ring cell carcinoma. Clin Pathol.

[3] Sciarra A, Uccella S, Hiroz P, Fournier I, Soubeyran V, Finzi G, La Rosa S (2023). Gastric amphicrine carcinoma showing neuroendocrine and pancreatic acinar cell differentiation. Lesson from a challenging case opening new perspectives in the diagnostic work-up of gastric neuroendocrine neoplasms. Endocr Pathol.

[4] Venturi S, Venturi A, Cimini D, Arduini C, Venturi M, Guidi A (1993). A new hypothesis: iodine and gastric cancer. Eur J Cancer Prev.

[5] Osborne D, Choudhary R, Vyas A, Kampa P, Abbas LF, Chigurupati HD, Alfonso M (2022). Hashimoto’s thyroiditis effects on papillary thyroid carcinoma outcomes: a systematic review. Cureus.

[6] Kandemir EG, Yonem A, Narin Y (2005). Gastric carcinoma and thyroid status. J Int Med Res.

[7] Wu XY, Liu WT, Wu ZF (2016). Identification of HRAS as cancer-promoting gene in gastric carcinoma cell aggressiveness. Am J Cancer Res.

[8] Dou R, Zhang L, Lu T, Liu D, Mei F, Huang J, Qian L (2018). Identification of a novel HRAS variant and its association with papillary thyroid carcinoma. Oncol Lett.

[9] Sun L, Wang C, Zhang J (2022). Genetic alterations in gastric amphicrine carcinomas and comparison with gastric mixed neuroendocrine-non-neuroendocrine neoplasms. Mod Pathol.

[10] Hitzeman N, Cotton E (2014). Incidentalomas: initial management. Am Fam Physician.

